# Supplementary material for: HIV-related challenges and women’s self-response: A qualitative study with women living with HIV in Indonesia
Source: PLoS One. 2022 Oct 10;17(10):e0275390. doi: 10.1371/journal.pone.0275390 (PMC9550025; doi:10.1371/journal.pone.0275390)
Supplement: Attachment — Submitted filename: renamed_d5dd8.docx [file pone.0275390.s002.docx]

Comments: HIV-related challenges and women’s self-response: a qualitative study with women living with HIV in Indonesia (PONE-D-22-13562)

This is an interesting paper. I have some comments as follows:

- Please explain what are PLHIV and WLHIV stands for in abstract as well as in the paper for the readers.
- Make sure you write succinctly – there are many sentences which are too long. For instance, on page 5, authors write: *“It is crucial to understand the lived experience of WLHIV themselves in order to effectively address the impact of the HIV epidemic at multiple levels, from immediate healthcare and mental health support to developing evidence-based programs and interventions that address the needs of WLHIV and increase their resilience and capacity to access these supports, and to driving government legislation and policy change.”*
- What is CD4 on page 5?
- Need to explain why authors collect data from “Yogyakarta city” only.
- Correct the grammar. For example:
  - On page 6, authors write: “One-on-one in-depth interviews were used to explore participants’ perceptions of factors leading to HIV infection, their lived experienced of HIV impact and of their **access HIV care services**.”
  - On page 7, authors write: This was followed by close coding to identify similar or redundant codes in order to reduce the **ling list** of code to a manageable number.
  - On page 10, authors write: These were reflected in others’ negative assumptions or attitudes towards them. For example, they were being suspected as a ‘naughty woman’ (means a sex worker or a woman **who has sex multiple men**), through which they acquired HIV infection:

There are others.

- On page 8, what do you mean by “single?” – is it never married?
- I wonder whether women’s and men’s HIV related experience are different? If so, how? I understand, authors only collected data from women, but if authors can explain or motivate on why only women, or how women’s experience can be different than men, that will be a good addition to the paper.
