# Supplementary material for: HIV-related challenges and women’s self-response: A qualitative study with women living with HIV in Indonesia
Source: PLoS One. 2022 Oct 10;17(10):e0275390. doi: 10.1371/journal.pone.0275390 (PMC9550025; doi:10.1371/journal.pone.0275390)
Supplement: Attachment — Submitted filename: Response to reviewers_plos1.docx [file pone.0275390.s003.docx]

**Response to Reviewers**

Dear Editor,

Thank you for considering our paper for publication in **PLOS ONE**. Herewith we submit the revised version of our paper entitled: **“HIV-related challenges and women’s self-response: a qualitative study with women living with HIV in Indonesia”**. We have improved the manuscript and addressed each of the comments from the reviewer as reported in the ‘a point-by-point response’ below.

On behalf of all authors,

Sincerely,

Hailay Abrha Gesesew

Journal Requirements:

When submitting your revision, we need you to address these additional requirements.

1. Please ensure that your manuscript meets PLOS ONE's style requirements, including those for file naming. The PLOS ONE style templates can be found at 
[https://journals.plos.org/plosone/s/file?id=wjVg/PLOSOne_formatting_sample_main_body.pdf](https://secure-web.cisco.com/128fX-HRgGQwl97gEkZBrvI-SrXvVhFpEKbD6Z_2bTNsSDwNiRKER1GCwPuAwEHzmCR9ZuLctnyFVXaPVCZqnQ3LURlnPc6lC4mZakXwbvnUNbaIS8-387UIHRidZy2Sa_F0wdR_1ds6zZkluy7ovfEzBll1GYEsuIFJfRaIws3FSNOBNMqZVZdtHo_avyZ1OfbBX3ECCdmZUIUCecITq_Qt36awimkB8fpnF4ZhD0x1SSMQ0IQWSYXzcnvMm_-3vYryGXssWJlI2268mN4k7G7DdMhSiZW1oaPhloh5KDA3cLiivg1Pg6h4lbJStHYHZ4kIEvJCK3p5xB2FmA9iDoQ/https%3A%2F%2Fjournals.plos.org%2Fplosone%2Fs%2Ffile%3Fid%3DwjVg%2FPLOSOne_formatting_sample_main_body.pdf) and 
[https://journals.plos.org/plosone/s/file?id=ba62/PLOSOne_formatting_sample_title_authors_affiliations.pdf](https://secure-web.cisco.com/1K3q9Nq_OVjHmLbzeD7H35nxh7GRBMc1M2xUJl-9rShlx1QbdAE2XaUq73mtoUTyCuNVyQ3w8UwTjm8c9MpUhVeZ44INbxDIvzCKZEXJZocB6YjVSOw-c9gmwWBNeg8B-DdI0BHFKPQaX6QK_75uRlGX1ZmaYVoKdfL9rX246FAmNoSQdVBzztF2wrUcvnS_sP2g7dzNIYOByQansDe9Yjhq3dWf4gYqT4f_Bt8Tx_x6gXsZFaUAOWvbH7CvlQ8gfex0woTnjbMBM48YUSf6eBMq9AMEysKI1BWKcg0T-dtb2ziBrvIuUlBl-nxpmg9VM1X_pZk1oMR-j9KJExvtxBQ/https%3A%2F%2Fjournals.plos.org%2Fplosone%2Fs%2Ffile%3Fid%3Dba62%2FPLOSOne_formatting_sample_title_authors_affiliations.pdf)

**Response**:

- Manuscript has been adjusted to PLOS ONE's style requirements

2. Please include a complete copy of PLOS’ questionnaire on inclusivity in global research in your revised manuscript. Our policy for research in this area aims to improve transparency in the reporting of research performed outside of researchers’ own country or community. The policy applies to researchers who have travelled to a different country to conduct research, research with Indigenous populations or their lands, and research on cultural artefacts. The questionnaire can also be requested at the journal’s discretion for any other submissions, even if these conditions are not met.  Please find more information on the policy and a link to download a blank copy of the questionnaire here: [https://journals.plos.org/plosone/s/best-practices-in-research-reporting](https://secure-web.cisco.com/1uYidGZkjftHb1-7OABCIAf88prap2t4k4PkmSDmBGqfyJvDLQPhgFRVf_nc8xkyElmortW3E74-JPEhWiun3JoxcUmr4uNNnWdqBua21AUaAF2Zj4MQKbna0aTmeqhccvvdg-Vj8BLM8zgcl_ueM0sY1FG8Uf4qkT837Hi77GPq69WTFxWCD1TTTwWdR7WXG8MN_c7iZECx-tFSHuFp2oC2PeXt5sNbofKVTVrepCVhc8DwSloK7r6wYeiIc_xRH_Q930j4ysxVEJ4fvLQtyHQUxWvvQS0JldP_Ir8CQnC2FoLD_YFiet1omUJU3o8b7b-T_5y-uPJVmZQsrloqWjw/https%3A%2F%2Fjournals.plos.org%2Fplosone%2Fs%2Fbest-practices-in-research-reporting). Please upload a completed version of your questionnaire as Supporting Information when you resubmit your manuscript.

**Response**

- PLOS’ questionnaire on inclusivity in global research is attached.
- We would like to explain that the first author (NKF) is from Indonesia, where the study was conducted. He was the field researcher who conducted the interviews with all the participants.

3. Please amend your current ethics statement to address the following concerns:
a) Did participants provide their written or verbal informed consent to participate in this study?
b) If consent was verbal, please explain i) why written consent was not obtained, ii) how you documented participant consent, and iii) whether the ethics committees/IRB approved this consent procedure.

**Response**

- Participants were provided with written informed consent form, signed and returned it to the researcher before commencing the interviews.

4. In your Data Availability statement, you have not specified where the minimal data set underlying the results described in your manuscript can be found. PLOS defines a study's minimal data set as the underlying data used to reach the conclusions drawn in the manuscript and any additional data required to replicate the reported study findings in their entirety. All PLOS journals require that the minimal data set be made fully available. For more information about our data policy, please see [http://journals.plos.org/plosone/s/data-availability](http://secure-web.cisco.com/1Gw3I8NPgQK35vo_3PRP6Ze7QJfZPf5i_jKfBSpp84Qv6pLi6GbUPTHxZkedw97h-hmV_ghGYc2vDfNkZQR3eAWtgNYyc3l1o3RT0ZVlUrZ83tYzpNyU4ljT7VHLPw7uWk6RmbaVORuv2PX42N5R69Am2tyTw2BW682pGebhy3sBaoBKlVSfAkKQkYK3fW8clppFOGCtGW5H3dPN6_qhlc-IHjZpsL2MNBWrKUck3OpbJkWIoMEWqeX7YEireVehXLolNkU-D6NfvecZqhXn6ZDoQGwx5A0Zk-gPhjjjtIFula2Ll_JGn-LnKUMnO-fCmJs7GmsCyRiWJeNTRwEH51Q/http%3A%2F%2Fjournals.plos.org%2Fplosone%2Fs%2Fdata-availability).

"Upon re-submitting your revised manuscript, please upload your study’s minimal underlying data set as either Supporting Information files or to a stable, public repository and include the relevant URLs, DOIs, or accession numbers within your revised cover letter. For a list of acceptable repositories, please see [http://journals.plos.org/plosone/s/data-availability#loc-recommended-repositories](http://secure-web.cisco.com/1FmQ3gRyHplMbZugJ_Mx98U54s5n8e4kIiLAivwldJAHIxS9Dqn4eSPrr_ghr1wuI_7g3BTolMFbDejJB8ei75_tRAB7cwrqmB9hGkTQO14hy1aCYpq6w48svoehP6uyfeASwPS_Fd8Pb3R3-TojFygZfzcIUVhlMEExzNmCyMoxubaM9zcvK4_M_8kRqBs5JyGikbixOqYAF0a55Flkvc0hQdab5e1oYeRs_PM6pRHM4ILfoJfmeD16kfz1kWmLO30etIQY86OdbgNN3k1xYbAItuBaJDmCgC1eDXXQqLue4C0DkygWQvpzVnfwTZeH5zmt94zYppoDyhB6cZcTVRw/http%3A%2F%2Fjournals.plos.org%2Fplosone%2Fs%2Fdata-availability%23loc-recommended-repositories). Any potentially identifying patient information must be fully anonymized.

Important: If there are ethical or legal restrictions to sharing your data publicly, please explain these restrictions in detail. Please see our guidelines for more information on what we consider unacceptable restrictions to publicly sharing data: [http://journals.plos.org/plosone/s/data-availability#loc-unacceptable-data-access-restrictions](http://secure-web.cisco.com/1nUjuQgyPBGG-AfrpQT9_N0Kh3cr1t1SdUfcPZVhg0uf9z9XhEuGKcPTcI0GG1pFLewopRzMYrm8OKZplpVARxu-d76077SQJdK8yo8mCK_7HOYGYHHJvcmQXzzslbxzQweRbdFF4A4OYmQ9vUFAO1Vu-AIsnqdJ-VCnHkjfKlElJiLL-l3a6dJaaoMoYRsdVI-fkmRbO_rdo6gM0-Z15H936FRf0SOo-RrHdUUpre2_BAXsI8Xcswt_U1si0XHNqeG51MI6is0d3yBCHuoE0-eTUU11uJjJ66dbEb3ZV9qNklSO2LLVn4SRVy952DBdbjxHQMwpZxDd4xGQnxby0gw/http%3A%2F%2Fjournals.plos.org%2Fplosone%2Fs%2Fdata-availability%23loc-unacceptable-data-access-restrictions). Note that it is not acceptable for the authors to be the sole named individuals responsible for ensuring data access.

We will update your Data Availability statement to reflect the information you provide in your cover letter.

**Response**

- Data availability statement:

We would like to explain that the data cannot be shared publicly because of restriction set by the human research ethics committees. Data presented in this study are available on request from the ethics committee imposing the restriction upon the data or from the corresponding author for researchers who meet the criteria for access to confidential data.

Our data in this study comprise transcriptions of digital audio recordings of each individual interview with women living with HIV and healthcare professionals in Yogyakarta, Indonesia. The interviews were focused on exploring their views and experiences about HIV-risk factors, their lived experiences of HIV impacts and barriers to access to HIV care services.

The nature of the restrictions upon the data is ethical due to the reason that participants (women living with HIV) involved in this study are considered by the ethics committee as highly vulnerable groups and data obtained from them may contain sensitive information.

The full name of the ethics committee imposing the restrictions upon the data is Social and Behavioural Research Ethics Committee, Flinders University, South Australia, Australia. For more information regarding ethical approval of the project please contact the Executive Officer on +618 8201-3116 or [human.researchethics@flinders.edu.au](mailto:human.researchethics@flinders.edu.au)

We revised the data availability statement as follows as per your suggestion.

“The data relevant to this study cannot be shared publicly as they contain sensitive and potentially identifying participant information. These restrictions were imposed by the Social and Behavioural Research Ethics Committee, Flinders University, South Australia, Australia. For more information regarding the ethical approval and for data access requests, please contact the Executive Officer at [human.researchethics@flinders.edu.au](mailto:human.researchethics@flinders.edu.au)”

5. Please review your reference list to ensure that it is complete and correct. If you have cited papers that have been retracted, please include the rationale for doing so in the manuscript text, or remove these references and replace them with relevant current references. Any changes to the reference list should be mentioned in the rebuttal letter that accompanies your revised manuscript. If you need to cite a retracted article, indicate the article’s retracted status in the References list and also include a citation and full reference for the retraction notice.

Additional Editor Comments:

Dear author your article is a good contribution to women with problems HIV, However is necessary to make minor improvements. I suggest you focused in methodology used. I suggest some reference that to aid

Zúniga-González, C. A., Jarquín-Saez, M. R., Martinez-Andrades, E., & Rivas-Garcia, J. A. (2016). Participatory Action Research: A Knowledge GenerationApproach (Investigación acción participativa: Un enfoque de generación del conocimiento). Rev. Iberoam. Bioecon. Cambio Clim., 2(1), 218–224.
**Response**

- This article has been cited.

**Review Comments to the Author**

Please use the space provided to explain your answers to the questions above. You may also include additional comments for the author, including concerns about dual publication, research ethics, or publication ethics. (Please upload your review as an attachment if it exceeds 20,000 characters)

**Reviewer #1:**

¬ As authors are required to make all data underlying the findings fully available without restrictions; please try and describe the data availability and the restrictions in detail. We understand that the authors are not willing to share the data due to its sensitive nature and keeping the identities of the study participants. However, please explain and justify why the data cannot be shared while maintaining anonymity or by using pseudonym for keeping the study participant’s identity secret.

**Response**

- We would like to explain that the data cannot be shared publicly because of restriction set by the human research ethics committees. Data presented in this study are available on request from the ethics committee imposing the restriction upon the data or from the corresponding author for researchers who meet the criteria for access to confidential data.

  Our data in this study comprise transcriptions of digital audio recordings of each individual interview with women living with HIV and healthcare professionals in Yogyakarta, Indonesia. The interviews were focused on exploring their views and experiences about HIV-risk factors, their lived experiences of HIV impacts and barriers to access to HIV care services.

The nature of the restrictions upon the data is ethical due to the reason that participants (women living with HIV) involved in this study are considered by the ethics committee as highly vulnerable groups and data obtained from them may contain sensitive information.

The full name of the ethics committee imposing the restrictions upon the data is Social and Behavioural Research Ethics Committee, Flinders University, South Australia, Australia. For more information regarding ethical approval of the project please contact the Executive Officer on +618 8201-3116 or [human.researchethics@flinders.edu.au](mailto:human.researchethics@flinders.edu.au)

¬ Please move the ‘Characteristics of the participants’ from the results to the ‘Methods’ section

**Response**

- ‘Characteristics of the participants’ has been integrated into the ‘Methods section’.

¬ It’s a good idea if the participants can be demonstrated in a table with some basic dynamics

**Response**

- A table containing characteristics of the participants has been provided.

| **Characteristics** | **Women living with HIV (n=26)** |
| --- | --- |
| **Age** |  |
| 20 - 29 | 6 |
| 30 - 39 | 12 |
| 40 - 49 | 8 |
| **Marital status** |  |
| Never married | 5 |
| Divorced/ Widowed | 8 |
| (Re)Married | 13 |
| **HIV diagnosis** |  |
| 1 - 5 years ago | 16 |
| 6 - 10 years ago | 7 |
| 11 - 15 years ago | 3 |
| **Religion** |  |
| Islam | 23 |
| Catholic | 2 |
| Protestant | 1 |
| **Education** |  |
| University graduate/Diploma | 6 |
| Senior High school graduate | 13 |
| Junior High school graduate | 6 |
| Elementary school graduate | 1 |
| **Occupation** |  |
| Housewife | 10 |
| Entrepreneur | 3 |
| Private employees (tailor, shopkeeper, banker, laundress, NGO worker) | 10 |
| Sex worker | 1 |
| University student | 1 |
| Unemployed | 1 |

¬ It’s a good idea to give a bit of narrative in between the quotations. Three or four quotations together is a bit tiring. Try to establish your argument using the quotations and put some narrative in between,

**Response**

- Thank you very much for this constructive suggestion, we have addressed this throughout the ‘Results section’.

¬ Collate typical and atypical responses and discuss accordingly

**Response**

- This has been addressed through the results and discussion sections.

¬ At the end of a section/paragraph, try to summarize the section in a couple of lines with some conclusive remarks. Rather than abruptly finishing a section with a couple of quotations.

**Response**

- We have provided a summary at the end of each section in the ‘Results section’.

¬ Please double-check your references and bibliography

**Response**

- We have checked and corrected the references

¬ Please do the formatting of the manuscript properly.

**Response**

- We have formatted the manuscript based on the journal guidelines

**Reviewer #2:** Comments: HIV-related challenges and women’s self-response: a qualitative study with women living with HIV in Indonesia (PONE-D-22-13562)

This is an interesting paper. I have some comments as follows:

• Please explain what are PLHIV and WLHIV stands for in abstract as well as in the paper for the readers.

**Response**

- These have been provided in the Abstract and the paper when they are used for the first time. PLHIV = People Living with HIV; and WLHIV = Women Living with HIV.

**In the abstract**:

HIV infection is a major public health concern, with a range of negative impacts on People Living with HIV (PLHIV). A qualitative study in Yogyakarta, Indonesia, using in-depth interviews with 26 Women Living with HIV (WLHIV) was conducted to understand HIV risk factors and impact and their access to HIV care services.

**In the body of the manuscript:**

Women Living with HIV (WLHIV) are…..

….. People Living with HIV (PLHIV) in 2021…

• Make sure you write succinctly – there are many sentences which are too long. For instance, on page 5, authors write: “*It is crucial to understand the lived experience of WLHIV themselves in order to effectively address the impact of the HIV epidemic at multiple levels, from immediate healthcare and mental health support to developing evidence-based programs and interventions that address the needs of WLHIV and increase their resilience and capacity to access these supports, and to driving government legislation and policy change.”*

**Response**

- We have revised this sentence and other long sentences.

• What is CD4 on page 5?

**Response**

- The services include providing health information on HIV and related services, HIV counselling and testing, cluster of differentiation 4 (CD4) and ….

• Need to explain why authors collect data from “Yogyakarta city” only.

**Response**

- To the best of our knowledge, there have never been studies that focused on exploring the self-response and capacity of PLHIV in Indonesia, and due to feasibility, familiarity and the potential to successfully conduct this study, Yogyakarta was chosen as the study setting.

• Correct the grammar. For example:
o On page 6, authors write: “One-on-one in-depth interviews were used to explore participants’ perceptions of factors leading to HIV infection, their lived experienced of HIV impact and of their access HIV care services.”

**Response**

- This has been revised:

In-depth interviews were used to explore participants’ perceptions of factors leading to HIV infection, their lived experiences of HIV impact, and their access to HIV care services.

o On page 7, authors write: This was followed by close coding to identify similar or redundant codes in order to reduce the ling list of code to a manageable number.

**Response**

- This has been revised:

This was followed by close coding to identify similar or redundant codes to reduce the long list of code to a manageable number.

o On page 10, authors write: These were reflected in others’ negative assumptions or attitudes towards them. For example, they were being suspected as a ‘naughty woman’ (means a sex worker or a woman who has sex multiple men), through which they acquired HIV infection:

**Response**

- This has been revised:

For example, they were being suspected as ‘naughty women’ or sex workers who have sex with multiple male partners, through which they acquired HIV infection.

There are others.
• On page 8, what do you mean by “single?” – is it never married?

**Response**

- ‘Never married’ is used.

• I wonder whether women’s and men’s HIV related experience are different? If so, how? I understand, authors only collected data from women, but if authors can explain or motivate on why only women, or how women’s experience can be different than men, that will be a good addition to the paper.

**Response**

- Although both women and men living with HIV have been reported to experience HIV-related psychological and social challenges, this report focuses on women due to the consideration, as reported in some previous studies in developing countries, including Indonesia, that WLHIV often experience a double burden due to the HIV diagnosis. Regardless of how they acquire the infection (e.g., through their spouses or partners) many WLHIV are often negatively labelled as ‘naughty women’ or women who have sex with multiple men, female sex workers, disloyal women, etc., which are not experienced by men living with HIV. Narratives of the women in this study were powerful and expressed experiencing psychological pressures, stigma and discrimination by their family members and other people around them following the HIV diagnosis. Despite experiencing these challenges, they demonstrated great self-response and capacity to pursue effective strategies and supports to protect themselves, and educate others around them to rebuild trust and respect. It is crucial to understand the lived experience of WLHIV themselves to effectively address the impact of the HIV epidemic at multiple levels. This may include government legislation and policy change and immediate healthcare and mental health support to developing evidence-based programs and interventions that address the needs of WLHIV and increase their resilience and capacity to access these supports.
